# Comparison of Intravenous Acetaminophen and Intravenous Patient-Controlled Analgesia Fentanyl after Total Hip Arthroplasty: A Multicenter Randomized Controlled Trial

**DOI:** 10.3390/jcm12237445

**Published:** 2023-11-30

**Authors:** Yoshinori Sakai, Norio Imai, Dai Miyasaka, Hayato Suzuki, Yoji Horigome, Yasuhito Takahashi, Hiroyuki Kawashima

**Affiliations:** 1 Department of Orthopedic Surgery, Niigata City General Hospital, 463-7 Shumoku, Chuouku, Niigata City 950-1197, Niigata, Japan; 2 Division of Comprehensive Musculoskeletal Medicine, Niigata University Graduate School of Medical and Dental Sciences, 1-757 Asahimachi-dori, Chuouku, Niigata City 951-8510, Niigata, Japan; 3 Department of Orthopedic Surgery, Niigata Bandai Hospital, 2-2-8 Yachiyo, Chuouku, Niigata City 950-8584, Niigata, Japan; 4 Division of Orthopedic Surgery, Department of Regenerative and Transplant Medicine, Niigata University Graduate School of Medical and Dental Sciences, 1-757 Asahimachi-dori, Chuouku, Niigata City 951-8510, Niigata, Japan; 5 Department of Orthopedic Surgery, Niigata Rosai Hospital, 1-7-12 Shinonome cho, Joetsu City 942-8502, Niigata, Japan

**Keywords:** total hip arthroplasty, patient-controlled analgesia, nausea and vomiting, acetaminophen, multimodal treatments, postoperative pain

## Abstract

Background: Opioids often need to be discontinued because they cause nausea, whereas the administration of intravenous acetaminophen (APAP) causes less nausea and vomiting. This study aimed to compare the effects of fentanyl-based intravenous patient-controlled analgesia (IV-PCA) and intravenous APAP on pain and nausea after total hip arthroplasty (THA). Methods: We prospectively investigated primary THA patients who underwent the anterolateral supine approach at four centers between October 2021 and October 2022. The patients (*n* = 178) were divided randomly into IV-PCA (*n* = 88) and APAP groups (*n* = 90). Rest pain, motion pain, and nausea were assessed using NRS scores. Results: Compared with the APAP group, the IV-PCA group experienced significantly greater resting pain and nausea on postoperative day 1. A correlation was found between preoperative and postoperative pain. Postoperative nausea at 8 h was significantly correlated with pain at rest at 4 h (r = 0.193), 8 h (r = 0.194), day 1 (r = 0.245), and day 2 (r = 0.188) after surgery. Early postoperative pain and nausea correlated with subsequent pain and nausea. Conclusions: Intravenous APAP is associated with less pain and nausea and is superior to IV-PCA.

## 1. Introduction

Intravenous patient-controlled analgesia (IV-PCA), including opioids, epidural anesthesia, spinal anesthesia, nerve blocks, nonsteroidal anti-inflammatory drugs (NSAIDs), and acetaminophen (APAP) is used for analgesia after total hip arthroplasty (THA) [1,2]. Although IV-PCA with opioids has excellent analgesic effects, several cases show nausea as a side effect [3,4,5]. Being unable to continue IV-PCA in cases where nausea and vomiting are often encountered is considered a weakness of this analgesic method.

Recently, the use of multimodal analgesia, which increases analgesic effects and reduces adverse effects through combining analgesics that act at multiple sites in the pain transmission pathway, has increased [2]. APAP causes less nausea and vomiting and is relatively safe [6,7]. Its concomitant use in post-THA pain management reduces narcotic use and side effects, including nausea and vomiting [8].

Pain is an anxiety factor for patients after undergoing THA; however, if postoperative pain is minimized, early rehabilitation can be performed effectively, leading to an earlier recovery of walking ability and the avoidance of adverse events, such as deep vein thrombosis [2]. Furthermore, the early recovery of hip function leads to shorter hospitalization and reduced medical costs [9,10]. Similarly, nausea and vomiting may cause patient dissatisfaction and disadvantages because of a lack of fluid and protein nutrients associated with decreased oral food intake, hindering early postoperative rehabilitation.

Most THA procedures in our groups are performed using a supine anterolateral approach. This minimally invasive surgery that preserves the muscles and tendons is considered less invasive and painful than conventional THA [11,12,13,14]. Therefore, postoperative pain management based on APAP without opioids has been speculated to provide sufficient analgesic effects and cause less nausea and vomiting than opioids.

Although multimodal analgesia has been shown to increase analgesic effects while decreasing side effects, few studies have compared the efficacy and side-effect profile of IV-PCA with those of opioids and intravenous APAP for pain management after THA. We hypothesized that postoperative pain management based on APAP would be an analgesic method with similar analgesic efficacy but less nausea and vomiting than in IV-PCA treatment. The aim of this study was to compare postoperative analgesia and side effects between intravenous APAP and IV-PCA fentanyl after total hip arthroplasty.

## 2. Materials and Methods

### 2.1. Participants

We prospectively investigated primary THA patients who underwent the anterolateral supine approach between October 2021 and October 2022 at four centers in Niigata Prefecture, Japan. The participants included male and female patients aged 30–80 years. The study was conducted in accordance with the Declaration of Helsinki and was approved by the Niigata University Ethics Committee (2021-0013). All research was performed in accordance with CONSORT guidelines/regulations, and written informed consent was obtained from the participants according to the study plan. This multicenter randomized controlled trial was registered at the Niigata University Ethics Committee on 20 September 2021 (UMIN-CTR ID: 000045480).

All patients were assigned to the IV-PCA or APAP group using a random-number table. Randomization was performed at each of the four facilities to avoid bias in the number of patients per facility between groups. Patients were excluded if they had liver disease, renal disease (estimated glomerular filtration rate < 30 mL/min/1.73 m^2^), a gastric ulcer, bronchial asthma, a psychiatric disease, or a research-related drug allergy. In addition, patients with a Crowe grade of 3–4 and intraoperative fractures and patients who had previously undergone hip joint or femur surgery were also excluded.

### 2.2. Study Protocol

All patients underwent surgery under general anesthesia. Intraoperative anesthesia was varied depending on the facility but standardized within the same facility. Moreover, whether the introduction and maintenance of anesthesia were via venous or inhalation routes depended on the facility.

In the IV-PCA group, patients were instructed to press the IV-PCA themselves or call the nurse and use the IV-PCA first when the pain was severe. IV-PCA administration was started at the end of surgery and continued until the day after surgery. IV-PCA contained fentanyl 0.4–0.7 μg/kg/h and droperidol. The total volume was 60 mL and started at 1 mL/h with a 1 mL bolus with a 10 min lockout time. If nausea persisted and was intolerable, IV-PCA was discontinued. However, even upon IV-PCA discontinuation, pain and nausea were continually evaluated until day 4. In the APAP group, 1000 mg APAP (15 mg/kg for patients weighing < 50 kg) was infused intravenously over 15 min immediately after surgery; the same dose was administered every 6 h until the next day.

In both groups, 40 mL of 0.375% ropivacaine (30 mL in patients weighing < 50 kg) was injected topically into the muscle layer and subcutaneous tissues before wound closure. Intravenous flurbiprofen, intramuscular ketoprofen and diclofenac suppositories were used for additional postoperative analgesia. Domperidone and Metoclopramide were used as antiemetics as needed. Celecoxib 400 mg/day and APAP 3000 mg/day were administered orally starting the day after surgery. The administration start times for APAP were adjusted so that APAP and IV-PCA fentanyl did not overlap. Tranexamic acid 1000 mg was administered intravenously at the beginning of the surgery and 6 h postoperatively [15].

### 2.3. Outcome Measurements

The collected survey items included patient age, sex, operative time, and total perioperative blood loss (hematocrit [Ht] values were used preoperatively and on postoperative day 7 and calculated using Gross’ formula) [16]. Resting pain, motion pain, and nausea were measured using a numerical rating scale (NRS) (0 = no pain or nausea; 10 = maximum imaginable pain or nausea) before surgery, upon return to the room (0 h), 4 h after surgery, 8 h after surgery, and on days 1, 2, and 4 after surgery. Resting pain was defined as “pain at rest in bed,” and motion pain was defined as “pain when moving from sitting to standing position”. In addition, data were collected on the number of times antiemetic drugs and additional analgesia were used until the day after surgery. For statistical analysis, patients were divided into two NRS-based groups: NRS of 0–3 (0 = none, 1–3 = mild pain and nausea) and NRS of 4 or higher (moderate to intolerable pain and nausea) [17,18].

Blood tests were performed at each time point (preoperative and postoperative days 1 and 3) to investigate the aspartate aminotransferase (AST) and alanine aminotransferase (ALT) levels. When the levels exceeded thrice the normal upper limit as defined by the respective institution’s standards (for example, if the normal upper limit was 35 U/L and the levels were above 105 U/L), APAP administration was promptly discontinued, and the patient was excluded from further evaluation. On postoperative day 7, a lower limb venous echocardiogram was performed by a laboratory technician to search for deep vein thrombosis (DVT). Postoperative therapy was allowed in both groups starting the day after surgery, with early loading according to pain.

### 2.4. Statistical Analysis

SPSS statistical software (version 28; SPSS, Inc., Chicago, IL, USA) was used to analyze the data. All patients were allocated to either the IV-PCA or APAP group based on a randomly generated number; the researchers generated a random allocation sequence. Each group was compared using the chi-squared test for categorical data and Student’s *t*-test for continuous variables. With regard to NRS, a Mann–Whitney U-test was conducted, using categorical data to compare APAP and IV-PCA group in each survey period. Pearson’s coefficient was used to evaluate the correlation between each NRS score. A chi-squared test was also conducted with the pain and nausea variables as follows: none/mild and moderate or higher. We have plotted the NRS as a boxplot with medians and interquartile range (IQR). A power analysis determined that the minimum number of cases for which the study could be validated with a power value > 0.8 was 88 cases per group. We also performed a post hoc analysis to evaluate statistical power (type II [β] error). We defined the effect size (d) as 0.5 and type I (α) error as 0.05 for the *t*-test, and the effect size (d) as 0.3 and type I (α) error as 0.05 in the chi-squared test. Differences were considered statistically significant at *p* < 0.05.

## 3. Results

Overall, 178 patients were included in the study; 88 were assigned randomly to the IV-PCA group and 90 to the APAP group. No significant differences were found in age, sex, body mass index (BMI), operative time, intraoperative blood loss, or total perioperative blood loss between the two groups (Table 1). Twelve patients (13.6%) in the IV-PCA group experienced severe nausea, and the analgesic treatment was discontinued prematurely.

**Figure 1:**
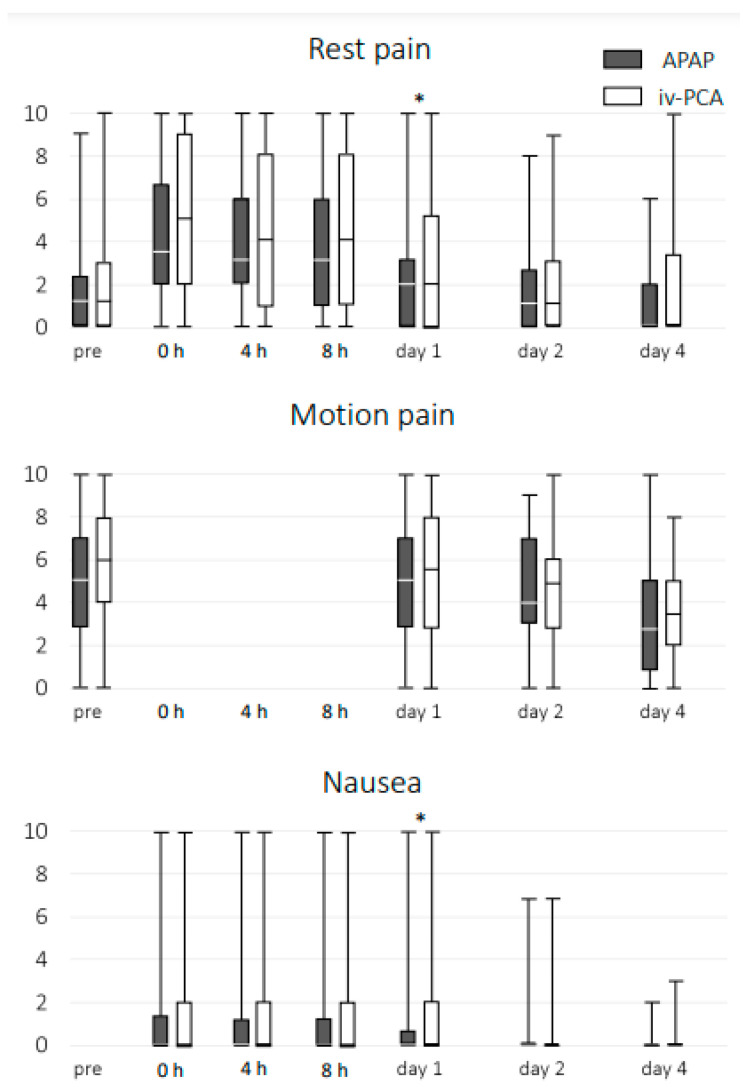
Numerical rating scale score graph of the groups. Resting pain on postoperative day 1 and nausea on postoperative day 1 were significantly higher in the IV-PCA group than in the APAP group. * Indicates statistical significance of *p* < 0.05. APAP, acetaminophen; IV-PCA, intravenous patient-controlled analgesia.

Resting pain on postoperative day 1 (*p* = 0.043) and nausea on postoperative day 1 (*p* = 0.047) were significantly greater in the IV-PCA group than in the APAP group (Figure 1). Moreover, moderate or severe resting pain was significantly greater in the IV-PCA group than in the APAP group on postoperative days 1 (*p* = 0.009) and 4 (*p* = 0.030), and nausea on postoperative day 1 (*p* = 0.041) (Table 2).

A correlation was found between preoperative and postoperative pain in all cases. No other preoperative factors were observed to influence postoperative nausea and pain (Table 3).

Nausea at 8 h postoperatively significantly correlated with rest pain at 4 h (r = 0.193) and 8 h (r = 0.194) and on day 1 (r = 0.245) and day 2 (r = 0.188) after surgery (Table 4). Other correlations between survey items are shown in table format in Supplementary Table S1.

No significant difference was observed in the number of times additional analgesia was used between the IV-PCA and APAP groups (36 times in the IV-PCA group and 28 times in the APAP group). Additionally, the administration of additional antiemetics was similar in the IV-PCA and APAP groups (14 times in the IV-PCA group and 12 times in the APAP group). Additional analgesia and additional antiemetics were more common in the IV-PCA group. DVT occurred in four (4.5%) and three (3.3%) patients in the IV-PCA and APAP groups, respectively, with no significant difference (*p* = 0.677). AST and ALT levels were abnormal in one patient in each group (1.14% and 1.11%, respectively; *p* = 0.987) (Table 5). The post hoc analysis revealed that the powers of the *t*-test, chi-squared test, and correlation were 0.913, 0.891, and 0.994, respectively.

## 4. Discussion

The study analysis showed that rest pain on postoperative day 1 and nausea on postoperative day 1 were significantly greater in the IV-PCA group compared to the APAP group. Moreover, moderate or severe resting pain was significantly greater in the IV-PCA group than in the APAP group on postoperative days 1 and 4, and nausea on postoperative day 1. Thus, APAP provided better analgesia for postoperative pain, with less postoperative nausea than IV-PCA. Moreover, APAP is effective in combination with NSAIDs, nerve blocks, and local injections in multimodal analgesia [8,19,20,21]. This study combined APAP with NSAIDs and local injection of ropivacaine, demonstrating high analgesic efficacy. Furthermore, the use of additional analgesics tended to be lower in the APAP group; most patients did not use any additional analgesics, suggesting that APAP alone is sufficient for postoperative analgesia in patients undergoing THA.

Roberts et al. found that opioid-induced postoperative nausea and vomiting occurred in 50% and 20% of their patients, respectively [3]. They also found a strong dose–response relationship between opioid use and postoperative nausea and vomiting. Particularly, postoperative nausea and vomiting increased as the dose increased for IV-PCA, with 70% of patients reporting nausea and 40% reporting vomiting [3]. Because nausea has disadvantages, such as discomfort, pain due to vomiting, elevated arterial pressure, increased blood loss, breathing problems, and wound dehiscence [22], analgesia with less nausea and vomiting effects is preferable. However, not all cases of postoperative nausea are opioid-induced. Several factors are involved, including the effects of intraoperative anesthesia, surgical invasion, and perioperative environment [4]. Wang et al. suggested that nausea and vomiting within 6 h after surgery might be caused by invasive procedures during surgery [23].

In this study, the NRS score of the IV-PCA group was significantly higher for nausea on postoperative day 1 than that of the APAP group. The NRS score of the IV-PCA group also tended to be higher 4 h postoperatively than that of the APAP group; however, the difference was not significant. Differences between the IV-PCA and APAP groups might have developed as the intraoperative effects decreased and opioid use increased. Furthermore, a significant correlation was found between nausea at 8 h postoperatively and pain at rest for approximately 2 days after surgery, suggesting that nausea may have influenced NRS scores for resting pain. These findings indicate that APAP, which causes less nausea and vomiting than IV-PCA, is preferable for postoperative analgesia.

Hepatic dysfunction is a potential side effect of APAP; however, only two liver function abnormalities were observed among all patients. In addition, although overdosage can be a problem, APAP is safe if the dosage is followed [7] and has not been a problem in other reports [6,8,20]. Consequently, we concluded that short-term drug use is not a problem.

This study has some limitations. First, the sample size was small; however, the sample size used was calculated to be the minimum number that a power test could validate. Second, all procedures were performed using an anterolateral supine approach, which preserves the muscles and tendons and may be more beneficial for functional recovery and less painful than the conventional approach [11,12,13,14]. Ukai et al. compared the early postoperative outcomes for postoperative pain and functional recovery between the anterolateral and posterolateral approaches, finding that the anterolateral approach was associated with a shorter time to beginning walking with a cane and a reduced hospitalization period. However, no significant differences were observed for pain [24]. Therefore, the influence of the approach on pain was considered small. Studies of other approaches, such as the posterolateral approach, produced results similar to those in the present study. Third, in this study, intraoperative anesthesia differed between four facilities, which may have affected the results. It was difficult to standardize anesthesia methods among the four facilities. Although the anesthesia method was standardized at each facility, we did not examine whether there were differences between facilities. However, randomization was performed at each of the four facilities to avoid bias between each group, and intraoperative anesthesia at each facility is thought to have little effect on the results. Fourth, the average age of the patients was 67 years old, so it is possible that PCA was not used properly. Older patients may have lower opioid tolerance and higher sensitivity to opioid adverse effects as well as cognitive impairments, poor vision, and limited manual dexterity that affects their ability to understand and use the device correctly. It cannot be ruled out that age may have influenced the results. However, we believe that this effect was small because patients were adequately instructed on how to use IV-PCA themselves or call the nurse when the pain was severe.

This study could not detect cases for which APAP or IV-PCA was suitable according to sex, BMI, or other factors. Therefore, further research is necessary to select an appropriate analgesic method for each patient.

## 5. Conclusions

Intravenous acetaminophen decreased the incidence of postoperative pain and nausea as compared to IV-PCA fentanyl and therefore may be the preferred analgesic choice for primary THA surgical patients.

## Figures and Tables

**Table 1:** Perioperative data for each group APAP, acetaminophen; IV-PCA, intravenous patient-controlled analgesia. * Student *t*-test; † chi-squared test.

	APAP (*n* = 90)	IV-PCA (*n* = 88)	*p*-Value
Age (years)	66.5 ± 8.2	67.9 ± 9.3	* 0.281
Sex (male/female)	18/72	20/66	† 0.600
Body mass index (kg/m^2^)	24.2 ± 4.2	23.9 ± 3.5	* 0.654
Operation time (min)	92.5 ± 20.1	89.8 ± 21.0	* 0.414
Intraoperative bleed (mL)	338.9 ± 147.5	346.7 ± 182.5	* 0.763
Estimated blood loss (mL)	899.9 ± 340.6	909.4 ± 370.1	* 0.864

**Table 2:** The number of participants experiencing moderate or severe pain and nausea in each group, according the numerical rating scale APAP, acetaminophen; IV-PCA, intravenous patient-controlled analgesia; RP, resting pain; MP, motion pain; N, nausea. * *p* < 0.05, chi-squared test.

	Pre		0 h		4 h		8 h		Day 1			Day 2			Day 4		
	RP	MP	RP	N	RP	N	RP	N	RP	MP	N	RP	MP	N	RP	MP	N
APAP	13	22	45	15	41	11	37	11	20	26	9	17	55	2	9	34	0
IV-PCA	14	21	52	14	46	16	45	19	32	29	19	26	52	5	17	43	0
*p*-value	0.537	0.928	0.223	0.891	0.370	0.163	0.179	0.095	0.009 *	0.557	0.034 *	0.097	0.736	0.247	0.030 *	0.175	/

**Table 3:** Correlation between preoperative and postoperative factors. RP, resting pain; MP, motion pain; N, nausea. * *p* < 0.05, Pearson’s coefficient.

	0 h		4 h		8 h		Day 1			Day 2			Day 4		
	RP	N	RP	N	RP	N	RP	MP	N	RP	MP	N	RP	MP	N
Age	−0.038	0.024	−0.198 *	−0.024	−0.071	−0.009	−0.080	−0.036	0.078	−0.122	−0.110	−0.168	−0.105	−0.049	−0.084
Body mass index	0.029	0.101	0.027	0.027	−0.021	0.038	−0.064	0.022	0.058	−0.031	−0.004	−0.033	−0.038	−0.026	0.155
Pre RP	0.292 *	0.201 *	0.306 *	0.048	0.352 *	0.094	0.457 *	0.237 *	−0.085	0.517 *	0.218 *	−0.035	0.472 *	0.211 *	0.001
Pre MP	0.192 *	−0.093	0.150	−0.071	0.167	−0.090	0.073	0.096	−0.090	0.106	0.095	−0.105	0.134	0.021	−0.091

**Table 4:** Correlation between rest pain and nausea after surgery. * *p* < 0.05, Pearson’s coefficient.

Rest Pain	0 h	4 h	8 h	Day 1	Day 2	Day 4
Nausea at 4 h	0.072	0.118	0.131	0.199 *	0.160	0.036
at 8 h	0.096	0.193 *	0.194 *	0.245 *	0.188 *	0.073

**Table 5:** Occurrence of DVT and elevated liver enzymes. APAP, acetaminophen; IV-PCA, intravenous patient-controlled analgesia; DVT, deep vein thrombosis.

	APAP (*n* = 90)	IV-PCA (*n* = 88)	*p*-Value
DVT	3 (3.3%)	4 (4.5%)	0.677
Elevated liver enzymes	1 (1.1%)	1 (1.1%)	0.987

## Data Availability

The datasets used and/or analyzed during the current study are available from the corresponding author upon reasonable request.

## Supplementary Materials

The following supporting information can be downloaded at: https://www.mdpi.com/article/10.3390/jcm12237445/s1, Table S1: Correlations between the survey items.
