# Supplementary material for: Comparison of Intravenous Acetaminophen and Intravenous Patient-Controlled Analgesia Fentanyl after Total Hip Arthroplasty: A Multicenter Randomized Controlled Trial
Source: J Clin Med. 2023 Nov 30;12(23):7445. doi: 10.3390/jcm12237445 (PMC10707402; doi:10.3390/jcm12237445)
Supplement: Supplementary file 1 [file jcm-12-07445-s001.zip › jcm-2743037-supplementary.pdf]

Supplementary Table S1. Correlations between the survey items

	Age	BMI	RP pre	MP pre	RP 0 h	N 0h	RP 4h	N 4h	RP 8h	N 8h	RP1d	MP1d	N1d	RP2d	M2d	N2d	RP4d	MP4d	N4d
Age		-0.018<br>0.818	-0.01<br>0.893	-0.001<br>0.985	-0.038<br>0.623	0.024<br>0.756	-0.138<br>0.074	-0.024<br>0.752	-0.071<br>0.362	-0.009<br>0.909	-0.08<br>0.299	-0.036<br>0.647	0.078<br>0.315	-0.122<br>0.115	-0.11<br>0.154	-0.168*<br>0.029	-0.105<br>0.174	-0.049<br>0.524	-0.084<br>0.277
BMI	-0.018<br>0.818		-0.1<br>0.206	-0.055<br>0.489	0.029<br>0.713	0.101<br>0.203	0.027<br>0.737	0.027<br>0.731	-0.021<br>0.795	0.038<br>0.633	-0.064<br>0.419	0.022<br>0.776	0.058<br>0.467	-0.031<br>0.699	-0.004<br>0.957	-0.033<br>0.681	-0.038<br>0.627	-0.026<br>0.745	0.155*<br>0.049
RP pre	-0.01<br>0.893	-0.1<br>0.206		0.227**<br>0.003	0.292**<br><.0.001	0.101<br>0.184	0.306**<br><0.001	0.048<br>0.526	0.352**<br><0.001	0.094<br>0.218	0.457**<br><0.001	0.237**<br>0.002	-0.085<br>0.265	0.517**<br><0.001	0.218**<br>0.004	-0.035<br>0.649	0.472**<br><0.001	0.211**<br>0.005	0.001<br>0.995
MP pre	-0.001<br>0.985	-0.055<br>0.489	0.227**<br>0.003		0.192*<br>0.011	-0.093<br>0.223	0.150*<br>0.048	-0.071<br>0.348	0.167*<br>0.027	-0.09<br>0.238	0.073<br>0.342	0.096<br>0.206	-0.09<br>0.237	0.106<br>0.165	0.095<br>0.212	-0.105<br>0.169	0.134<br>0.078	0.021<br>0.778	-0.091<br>0.235
RP 0h	-0.038<br>0.623	0.029<br>0.713	0.292**<br><.0.001	0.192*<br>0.011		0.076<br>0.316	0.688**<br><0.001	0.072<br>0.346	0.665**<br><0.001	0.096<br>0.207	0.511**<br><0.001	0.429**<br><0.001	0.03<br>0.693	0.405**<br><0.001	0.373**<br><0.001	-0.068<br>0.372	0.355**<br><0.001	0.265**<br><0.001	-0.049<br>0.524
N 0h	0.024<br>0.756	0.101<br>0.203	0.101<br>0.184	-0.093<br>0.223	0.076<br>0.316		0.096<br>0.221	0.613**<br><0.001	0.094<br>0.215	0.604**<br><0.001	0.133<br>0.081	0.071<br>0.349	0.300**<br><0.001	0.081<br>0.289	0.1<br>0.189	0.319**<br><0.001	0.088<br>0.25	0.074<br>0.329	0.356**<br><0.001
RP4h	-0.138<br>0.074	0.027<br>0.737	0.306**<br><0.001	0.150*<br>0.048	0.688**<br><0.001	0.096<br>0.221		0.118<br>0.121	0.948**<br><0.001	0.173*<br>0.023	0.720**<br><0.001	0.613**<br><0.001	0.053<br>0.488	0.504**<br><0.001	0.484**<br><0.001	0.089<br>0.241	0.481**<br><0.001	0.398**<br><0.001	0.075<br>0.327
N 4h	-0.024<br>0.752	0.027<br>0.731	0.048<br>0.526	-0.071<br>0.348	0.072<br>0.346	0.613**<br><0.001	0.118<br>0.121		0.131<br>0.085	0.961**<br><0.001	0.199**<br><0.001	0.1<br>0.19	0.463**<br><0.001	0.160*<br>0.035	0.135<br>0.077	0.443**<br><0.001	0.036<br>0.633	0.052<br>0.496	0.396**<br><0.001
RP 8h	-0.071<br>0.362	-0.021<br>0.795	0.352**<br><0.001	0.167*<br>0.027	0.665**<br><0.001	0.094<br>0.215	0.948**<br><0.001	0.131<br>0.085		0.191*<br>0.011	0.742**<br><0.001	0.629**<br><0.001	0.072<br>0.346	0.156**<br><0.001	0.486**<br><0.001	0.072<br>0.348	0.486**<br><0.001	0.433**<br><0.001	0.072<br>0.344
N 8h	-0.009<br>0.909	0.038<br>0.633	0.094<br>0.218	-0.09<br>0.238	0.096<br>0.207	0.604**<br><0.001	0.173*<br>0.023	0.961**<br><0.001	0.191*<br>0.011		0.245**<br><0.001	0.154*<br>0.043	0.476**<br><0.001	0.188*<br>0.013	0.170*<br>0.025	0.454**<br><0.001	0.073<br>0.341	0.108<br>0.157	0.421**<br><0.001
RP1d	-0.08<br>0.299	-0.064<br>0.419	0.457**<br><0.001	0.073<br>0.342	0.511**<br><0.001	0.133<br>0.081	0.720**<br><0.001	0.199**<br><0.001	0.742**<br><0.001	0.245**<br><0.001		0681**<br><0.001	0.147<br>0.053	0.709**<br><0.001	0.524**<br><0.001	0.172*<br>0.023	0.656**<br><0.001	0.466**<br><0.001	0.086<br>0.258
MP1d	-0.036<br>0.647	0.022<br>0.776	0.237**<br>0.002	0.096<br>0.206	0.429**<br><0.001	0.071<br>0.349	0.613**<br><0.001	0.1<br>0.19	0.629**<br><0.001	0.154*<br>0.043	0681**<br><0.001		0.132<br>0.082	0.495**<br><0.001	0.760**<br><0.001	0.122<br>0.11	0.515**<br><0.001	0.612**<br><0.001	0.03<br>0.691
N1d	0.078<br>0.315	0.058<br>0.467	-0.085<br>0.265	-0.09<br>0.237	0.03<br>0.693	0.300**<br><0.001	0.053<br>0.488	0.463**<br><0.001	0.072<br>0.346	0.476**<br><0.001	0.147<br>0.053	0.132<br>0.082		0.102<br>0.181	0.076<br>0.321	0.470**<br><0.001	0.029<br>0.704	-0.006<br>0.94	0.295**<br><0.001
RP2d	-0.122<br>0.115	-0.031<br>0.699	0.517**<br><0.001	0.106<br>0.165	0.405**<br><0.001	0.081<br>0.289	0.504**<br><0.001	0.160*<br>0.035	0.156**<br><0.001	0.188*<br>0.013	0.709**<br><0.001	0.495**<br><0.001	0.102<br>0.181		0.644**<br><0.001	0.284**<br><0.001	0.813**<br><0.001	0.590**<br><0.001	0.117<br>0.123
MP2d	-0.11<br>0.154	-0.004<br>0.957	0.218**<br>0.004	0.095<br>0.212	0.373**<br><0.001	0.1<br>0.189	0.484**<br><0.001	0.135<br>0.077	0.486**<br><0.001	0.170*<br>0.025	0.524**<br><0.001	0.760**<br><0.001	0.076<br>0.321	0.644**<br><0.001		0.156*<br>0.04	0.565**<br><0.001	0.756**<br><0.001	0.031<br>0.683
N2d	-0.168*<br>0.029	-0.033<br>0.681	-0.035<br>0.649	-0.105<br>0.169	-0.068<br>0.372	0.319**<br><0.001	0.089<br>0.241	0.443**<br><0.001	0.072<br>0.348	0.454**<br><0.001	0.172*<br>0.023	0.122<br>0.11	0.470**<br><0.001	0.284**<br><0.001	0.156*<br>0.04		0.109<br>0.151	0.14<br>0.066	0.680**<br><0.001
RP4d	-0.105<br>0.174	-0.038<br>0.627	0.472**<br><0.001	0.134<br>0.078	0.355**<br><0.001	0.088<br>0.25	0.481**<br><0.001	0.036<br>0.633	0.486**<br><0.001	0.073<br>0.341	0.656**<br><0.001	0.515**<br><0.001	0.029<br>0.704	0.813**<br><0.001	0.565**<br><0.001	0.109<br>0.151		0.638**<br><0.001	0.004<br>0.958

MP4d	-0.049<br>0.524	-0.026<br>0.745	0.211**<br>0.005	0.021<br>0.778	0.265**<br><0.001	0.074<br>0.329	0.398**<br><0.001	0.052<br>0.496	0.433**<br><0.001	0.108<br>0.157	0.466**<br><0.001	0.612**<br><0.001	-0.006<br>0.94	0.590**<br><0.001	0.756**<br><0.001	0.14<br>0.066	0.638**<br><0.001		0.054<br>0.48
N4d	-0.084<br>0.277	0.155*<br>0.049	0.001<br>0.995	-0.091<br>0.235	-0.049<br>0.524	0.356**<br><0.001	0.075<br>0.327	0.396**<br><0.001	0.072<br>0.344	0.421**<br><0.001	0.086<br>0.258	0.03<br>0.691	0.295**<br><0.001	0.117<br>0.123	0.031<br>0.683	0.680**<br><0.001	0.004<br>0.958	0.054<br>0.48	

** $p < 0.05$: Pearson’s coefficient

BMI, body mass index; RP, resting pain; MP, motion pain; N, nausea; pre, preoperatively; 0h, 0 hours; 4h, 4 hours; 8h, 8 hours; 1d, day 1; 2d, day 2; 4d, day 4
